# Reviewer acknowledgement 2014

**DOI:** 10.1186/s13030-015-0033-5

**Published:** 2015-02-12

**Authors:** Koji Tsuboi

**Affiliations:** Department of Psychosomatic Medicine, Toho University School of Medicine, Tokyo, Japan

## Abstract

The *BioPsychoSocial Medicine* editorial team would like to thank the following colleagues who contributed to peer review for the journal in Volume 8 (2014).

**Mahmoud AL-Omiri**

Jordan

**Marie Amitani**

Japan

**Tetsuya Ando**

Japan

**Shuji Aou**

Japan

**Akihiro Asakawa**

Japan

**Takanobu Baba**

Japan

**Vassilis Barkoukis**

Greece

**Ananda Balayogi Bhavanani**

India

**Bruno Bonaz**

France

**Isabelle Bray**

United Kingdom

**Marc Cohen**

Australia

**Yukihiko Fujita**

Japan

**Shin Fukudo**

Japan

**Mikihiko Fukunaga**

Japan

**Toyohiro Hamaguchi**

Japan

**Makoto Hashiro**

Japan

**Makoto Hashizume**

Japan

**Peter Herschbach**

Germany

**Taina Hintsa**

Finland

**Reiko Hori**

Japan

**Bjoern Horing**

United States of America

**Mizuho Hosogi**

Japan

**Lai Ming Christy Hui**

Hong Kong

**Akio Inui**

Japan

**Yuko Ishizaki**

Japan

**Tsutomu Kamei**

Japan

**Kenji Kanbara**

Japan

**Michiko Kano**

Japan

**Norito Katoh**

Japan

**Keisuke Kawai**

Japan

**Kenji Kawakita**

Japan

**Monika Keller**

Germany

**Hiroe Kikuchi**

Japan

**Masayo Kojima**

Japan

**Hiroaki Kumano**

Japan

**Hubert Lacey**

United Kingdom

**Richard Lane**

United States of America

**Ichiro Mashima**

Japan

**Jason Mazanov**

Australia

**Christian Montag**

Germany

**Astrid Müller**

Germany

**Takao Munemoto**

Japan

**Tomohiko Muratsubaki**

Japan

**Yoshikatsu Nakai**

Japan

**Mutsuhiro Nakao**

Japan

**Nobuo Nishi**

Japan

**Shinobu Nomura**

Japan

**Takehiro Nozaki**

Japan

**Yuko Odagiri**

Japan

**Daisuke Ohta**

Japan

**Takakazu Oka**

Japan

**Gaby Resmark**

Germany

**Annelieke Roest**

Netherlands

**Hironori Shimada**

Japan

**Tomotaka Shoji**

Japan

**Katsuhisa Sunada**

Japan

**Shu Takakura**

Japan

**Takeaki Takeuchi**

Japan

**Yoshiyuki Takimoto**

Japan

**Hidetaka Tanaka**

Japan

**Jun Tayama**

Japan

**Yuri Terasawa**

Japan

**Daniel Widmer**

Switzerland

**Jong Min Woo**

Korea, South

**Jumpei Yajima**

Japan

**Kosuke Yamada**

Japan

**Kazufumi Yoshihara**

Japan

**Kazuhiro Yoshiuchi**

Japan

**Stephan Zipfel**

Germany

